# Quantitative Structure-Activity Relationship Studies of 4-Imidazolyl- 1,4-dihydropyridines as Calcium Channel Blockers

**Published:** 2013-08

**Authors:** Farzin Hadizadeh, Saadat Vahdani, Mehrnaz Jafarpour

**Affiliations:** 1 Biotechnology Research Center, Mashhad University of Medical Sciences, Mashhad, Iran; 2 Department of Chemistry, Islamic Azad University-North Tehran Branch, Tehran, Iran; 3 School of Pharmacy, Shiraz University of Medical Sciences, Shiraz, Iran

**Keywords:** Dihydropyridines, Genetic algorithm, MLR, pIC_50_, QSAR

## Abstract

***Objective(s): ***The structure- activity relationship of a series of 36 molecules, showing L-type calcium channel blocking was studied using a QSAR (quantitative structure–activity relationship) method.

***Materials and Methods: ***Structures were optimized by the semi-empirical AM1 quantum-chemical method which was also used to find structure-calcium channel blocking activity trends. Several types of descriptors, including electrotopological, structural and thermodynamics were used to derive a quantitative relationship between L-type calcium channel blocking activity and structural properties. The developed QSAR model contributed to a mechanistic understanding of the investigated biological effects.

***Results:*** Multiple linear regressions (MLR) was employed to model the relationships between molecular descriptors and biological activities of molecules using stepwise method and genetic algorithm as variable selection tools. The accuracy of the proposed MLR model was illustrated using cross-validation, and Y-randomisation -as the evaluation techniques.

***Conclusion:*** The predictive ability of the model was found to be satisfactory and could be used for designing a similar group of 1,4- dihydropyridines , based on a pyridine structure core which can block calcium channels.

## Introduction

Voltage-gated calcium channels are transmembrane proteins which allow selective Ca^2+ ^permeation in excitable cells, upon membrane depolarization. Voltage-gated calcium channels are heteromeric proteins consisting of the pore forming a1 subunit, disulfide-linked transmembrane complex of a2 and d subunits, intracellular b subunit and a subunit characteristic for skeletal muscle Ca^2+^ channels (1). Variability of regularity subunits distinguishes the tissue-specific calcium channel types L, N, T, P, Q and R (2). L-type Ca^2+^ channels are sensitive to numerous agonist and antagonist drugs that modulate the Ca^2+^ flow. Dihydropyridines (DHP) include both blocker and activators of L-type Ca^2+^ channels (3). Since their introduction as calcium channel blockers by Fleckenstein (4), these compounds have achieved special significance in the therapy of hypertension, angina pectoris and cardiovascular disease (5). Among the classes of calcium channel blockers, DHP derivatives are widely used. A quantitative structure–activity relationship (QSAR) study indicated that the potency of nifedipine analogues was dependent upon lipophilicity and electronic term and separate terms for each position on the aromatic ring (6). Making changes in the substitution pattern at C-3, C-4 and C-5 positions of nifedipine alter its potency (7), tissue selectivity (8, 9) and conformation of the 1,4-dihydropyridine ring (10). Our previous studies suggested that heterocyclic substituent like 1-substituted - alkylthioimidazol - 5-yl as bioisosteric replacement of nitrophenyl group at C-4 , enabled these compounds to have potent calcium antagonist activity (11-14). QSAR analysis is an effective method in the field of designing rational drugs and discovering the mechanism of drug actions. The fundamental hypothesis of the QSAR methodology is that the biological activity is a function of the molecular structure. 

This method is used to find empirical relationships in a set of compounds (the instructional set) that are known to have interesting properties. Here, calcium channel blocking activity was the biological effect investigated. Such an approach to study the SAR consists of three basic stages. These are forming the instructional (investigational) set of compounds and selecting the descriptors. In addition, it is useful in areas like designing virtual compound libraries and optimizing computational-chemical of compounds. QSAR studies can express the biological activities of compounds as a function of their various structural parameters and also describes how the variation in biological activity depends on changes in the chemical structure (15). Recently, a QSAR study of biological activity has been published by our research team (16-18). If such a relationship can be derived from the structure-activity data, the model equation allows medicinal chemists conclude with an agreeable degree of confidence which properties are determing in the mechanism of drug action. The success of a QSAR study depends on choosing robust statistical methods for producing the predictive model and also the relevant structural parameters for expressing the essential features within those chemical structures. Nowadays, genetic algorithms (GA) are well known as interesting and widely used methods for variable selection (19-25). GA are stochastic methods used to solve the optimization problems defined by the fitness criteria, applying the evolutionary hypothesis of Darwin and also different genetic functions i.e. crossover and mutation. In the present work, we have used a genetic algorithm for the variable selection and developed an MLR model for the QSAR analysis of the 1, 4- dihydropyridines compounds. In a QSAR study, the model must be validated for its predictive value before it can be used to predict the response of additional chemicals. Validating QSAR with external data (i.e. data not used in the model development), although demanding, is the best method for validation. Finally, the accuracy of the proposed model was illustrated using leave one out, cross-validations and Y-randomisation techniques.

## Materials and Methods

***Data set***

In this study, the data set of 1,4- dihydropyridines constitutes a group of small organic compounds based on a core pyridine structure which can both block and enhance calcium currents. (10-12). The inhibitory activity values are expressed as the half maximal inhibitory concentration (IC_50_). The chemical structures and activity data for the complete set of compounds are presented in Table 1. The activity data [IC_50_ (μM)] was converted to the logarithmic scale pIC_50_ [-log IC_50_ (M)] and then used for the subsequent QSAR analyses as the response variables (26). The data set was randomly divided into two subsets: the training set containing 29 compounds (80%) and the test set containing 7 compounds (20%). The training set was used to build a regression model and the test set was used to evaluate the predictive ability of the obtained model. 

***Structure entry and optimization***

All of the molecules were drawn into the HyperChem software (Version 7.0 Hypercube, Alberta, Canada) and pre-optimized using the MM+ molecular mechanics force field. Then, a more precise optimization was performed with the semi-empirical AM1 method in HyperChem (27). The molecular structures were optimized using the Polak–Ribiere algorithm until the root mean square gradient reached 0.01.

***Molecular descriptor generation***

 The Dragon packages (28) were used for calculating the molecular descriptors. The molecular structures were saved by the HIN extension and entered in the DRAGON software for the calculation of the 18 different types of theoretical descriptors for each molecule. They included (a) 0D-constitutional (atom and group counts); (b) 1D-functional groups, 1D-atom centered fragments; (c) 2D-topological, 2DBCUTs, 2D-walk and path counts, 2D-autocorrelations, 2D-connectivity indices, 2D-information indices, 2D-topological charge indices, and 2D-eigenvalue-based indices; and (d) 3D-Randic molecular profiles from the geometry matrix, 3D-geometrical, 3D-WHIM, and 3D-GETAWAY descriptors. These descriptors could represent a variety of aspects of the compounds and have been successfully used in various QSAR and quantitative structure-property relationship (QSPR) researches. Any descriptors with a constant or almost constant value for all the molecules were eliminated. Also, any pairs of variables with a correlation coefficient greater than 0.90 were classified as inter-correlated and only one of them were considered in developing the model. A total of 557 descriptors were considered for further investigations after discarding the descriptors with constant values and the ones that were inter-correlated. 

***Genetic algorithm***

Genetic algorithms (GAs) are governed by biological evolution rules (29). These are stochastic optimization methods that have been inspired by evolutionary principles. The distinctive aspect of a GA is that it investigates many possible solutions, simultaneously, each of which explores a different region in the parameter of space (30). To select the most relevant descriptors, the evolution of the population was simulated (31, 32). The first generation population was randomly selected; each individual member in the population was defined by a chromosome of binary values and represented a subset of descriptors. The number of the genes at each chromosome was equal to the number of the descriptors. A gene was given the value of 1, if its corresponding descriptor was included in the subset; otherwise, it was given the value of zero. The number of genes with the value of 1 was kept relatively low to have a small subset of descriptors (33). The genetic algorithm used in this paper is an evolution of the algorithm described in the reference #34, from which, parameters are reported in Table 2. Each wavelength subset selected in the spectrum will be represented by a p-dimensional vector and w, with binary coordinates. If the *ith *wavelength is selected then the *ith* coordinate of w is one, otherwise it is considered as  zero. Each w is a chromosome. Given a chromosome (w), a MLR calibration is constructed using, from each spectrum, only the wavelengths represented by w. Each chromosome is evaluated using the PRESS (w) value reached in the calibration. The genetic algorithm searches for the minimum PRESS (w) in the space of all the possible chromosomes without establishing, a priori, the latent structure of the calibration.

**Table1:** Chemical structures and the corresponding observed and predicted pIC_50_ values as measured by the MLR method

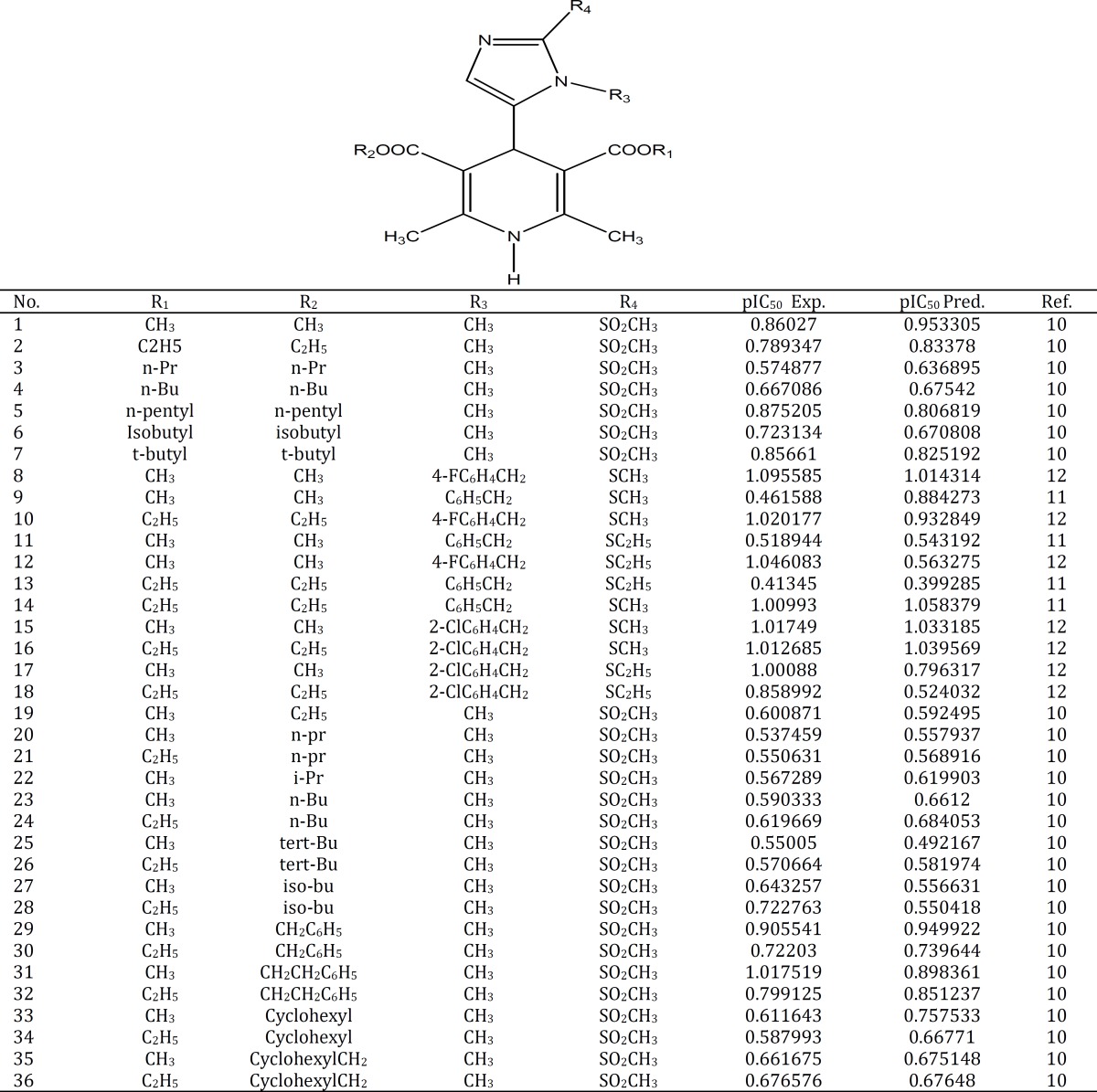

**Table 2:** Parameters of the genetic algorithm

Population size: 30 chromosomes
In average, five variables per chromosome in the original
population
Regression method: PLS
Response: cross-validated % explained variance (five deletion
groups; the number of components is determined by cross
validation)
Maximum number of variables selected in the same
chromosome: 30
Probability of mutation: 1%
Probability of crossover: 50%

**Figure 1:**
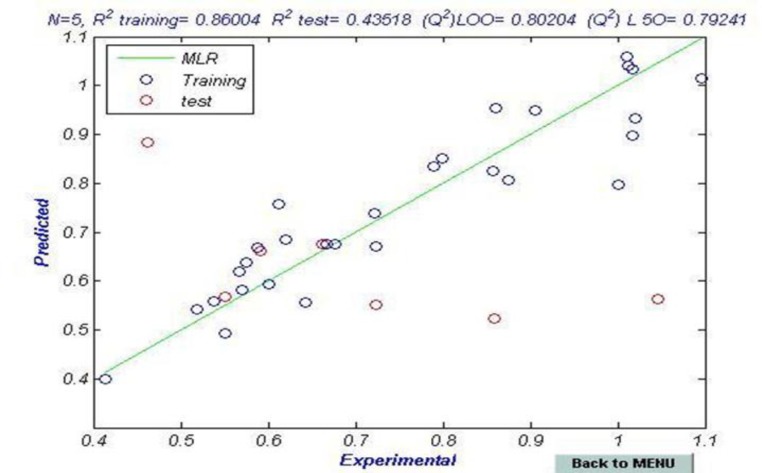
The predicted versus the experimental pIC_50_ measured by GA-MLR

**Figure 2:**
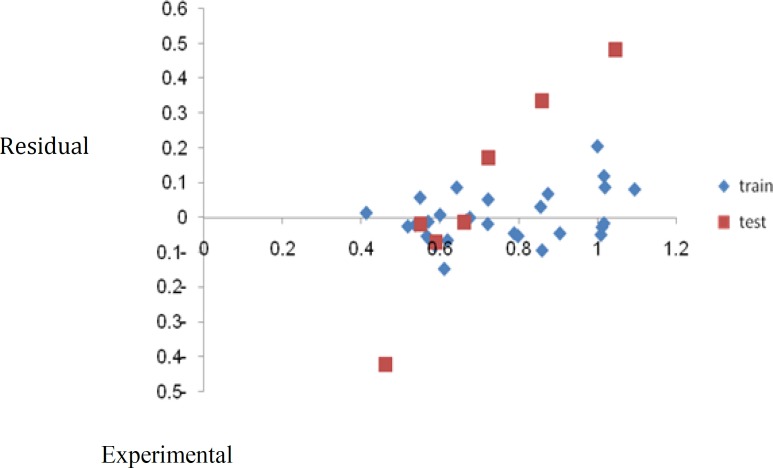
The residual versus the experimental pIC_50_ by measured GA-MLR

## Results

 In a QSAR study, generally, the quality of a model is expressed by its fitting ability and prediction ability, from which the latter is more important. With the selected descriptors, we have built a linear model using the set data and the following equation was obtained.

pIC_50_= -2.1301(0.951352)+1.457617(0.585674) BELm6 -1.08595(0.233582) E1m -2.25419(0.369137)E2v+3.7547(1.007998) HATS8m+19.65472(4.570518) R2e+                    (1)

N_train_=36 N_test_=7 R^2^_train_= 0.86 R^2^_test_=0.435

R^2^_adj_=0.830 F_train_=28.26 F_test_= 0.04 Q^2^_LOO_=0.802 

Q^2^_LGO_=0.792 Q^2^_BOOT_=0.796 RMSE _train_: 0.0715 RMSE _test_: 0.2826 

 In this equation, N is the number of compounds, R^2^ is the squared correlation coefficient, Q^2^_LOO_, Q^2^_BOOT_ and Q2_LGO_ are the squared cross-validation coefficients for leave one out, bootstrapping and external test set, respectively, RMSE is the root mean square error and F is the Fisher F statistic. The figures in parentheses are the standard deviations. The built model was used to predict the test set data and the prediction results are given in Table 1. As it is seen in Table 1, calculated values for the pIC_50_ are in good agreement with those of the experimental values. The predicted values for pIC_50_ for the compounds in the training and test sets using equation 1 were plotted against the experimental pIC_50_ values in Figure 1. A plot of the residual for the predicted values of pIC_50_ for both training and test sets against the experimental pIC_50_values are shown in Figure 2. Clearly, the model did not show any proportional and systematic error, because the propagation of the residuals on both sides of zero is random. The real usefulness of QSAR models is not just their ability to reproduce known data verified by their fitting power (R^2^), but mainly it is their predictive application potential . 

**Figure 3:**
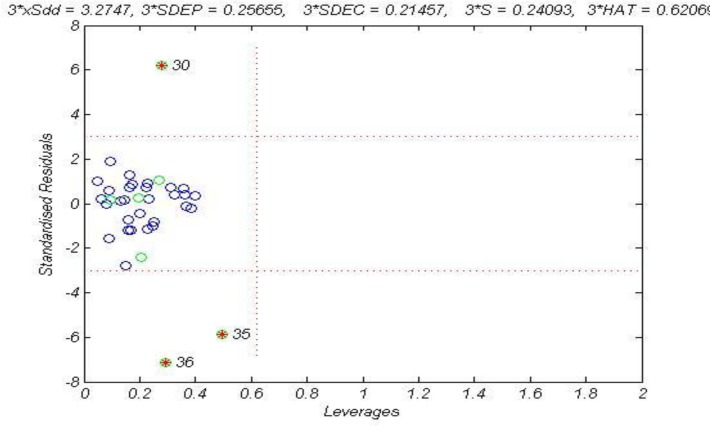
The William plot of the GA-MLR model

For this reason, the model calculations were performed by maximizing the explained variance in prediction verified by the leave-one-out cross-validated correlation coefficient (Q^2^_LOO_). To avoid the risk of over fitting and the possibility of overestimating the model predictivity by using Q^2^_LOO_, and Q^2^_LGO_, the internal predictive ability of the models was also verified using the bootstrap Q^2^_BOOT_ procedure, as is strongly recommended for QSAR modeling. The robustness of the proposed models and their predictive ability was guaranteed by the high Q^2^_BOOT_ based on the bootstrapping being repeated 5000 times. The Q^2^_LOO_, Q^2^_LGO _and Q^2^_BOOT_ for the MLR model are shown in equation 1. This indicates that the obtained regression model has a good internal and external predictive power. Also, in order to assess the robustness of the model, the Y-randomization test was applied in this study. The dependent variable vector (pIC_50_) was randomly shuffled and a new QSAR model developed using the original independent variable matrix. The new QSAR models (after several repetitions) would be expected to have low R^2^ and Q^2^_LOO_ values Table 3. If the opposite happens, acceptable QSAR model cannot be obtained for the specific modeling method and data.

The Williams plot (Figure 3), the plot of the standardized residuals versus the leverage, was exploited to visualize the applicability domain (36). The leverage indicates a compound’s distance from the centroid of X. The leverage of a compound in the original variable space is defined as (37, 38). 

$h_{i}=x_{i}^{T}{\left(X^{T}\right)}^{-1}x_{i}$                     (1)

Where *xi *is the descriptor vector of the considered compound and X is the descriptor matrix derived from the training set descriptor values. The warning leverage (h*) is defined as:

$h^{*}=\frac{3p}{n}$

Where *n* is the number of calibration compounds, *p* is the number of model variables plus one. The leverage (*h*) greater than the warning leverage (h*) suggested that the compound was very influential on the model. 

The MLR analysis was employed to derive the QSAR models for different 1, 4- dihydropyridines. MLR and correlation analyses were carried out by the statistics software SPSS (Version 16.0) Table 4.

**Table 3:** The R^2^ train and Q^2^_LOO _values after several Y-randomisation tests

No	Q^2^	R^2^
1	0.019534	0.287701
2	0.000557	0.242321
3	1.79E-05	0.228966
4	0.047437	0.119975
5	0.000167	0.19419
6	0.316241	0.060208
7	0.026796	0.127187
8	0.141785	0.09585
9	0.19683	0.063782
10	0.000406	0.215877

## Discussion

After analyzing we spillted the data set into the training set and test set, the next step was to select the main factors which were the most important for the L-type calcium channel blocking inhibition activity of of 1,4- dihydropyridines. As we do not know yet which descriptors or which particular combinations are related to the studied response and can be used in the predictive models, we applied genetic algorithms as the variable selection procedure to select only the best combinations (most relevant) for obtaining the models with the highest predictive power by using the training set. Five most significant descriptors according to the GA-MLR algorithm are lowest eigenvalue n. 6 of Burden matrix / weighted by atomic masses (BELm6), 1st component accessibility directional WHIM index / weighted by mass (E1m), 2nd component accessibility directional WHIM index / weighted by van der Waals volume (E2v), leverage-weighted autocorrelation of lag 8/weighted by mass (HATS8m) and R maximal autocorrelation of lag 2 / weighted by Sanderson electronegativity (R2e+).

The multi-collinearity between the above five descriptors were detected by calculating their variation inflation factors (VIF), which can be calculated as follows.

$\mathrm{VIF}=\frac{1}{1-\mathrm{r2}}$                      (2)

Where r is the correlation coefficient of the multiple regression between the variables in the model. If VIF equals 1, no inter-correlation exists for each variable; if VIF falls into the range of 1–5, the related model is acceptable; and if VIF is larger than 10, the related model is unstable and a recheck is necessary (39). The corresponding VIF values of the seven descriptors are shown in Table 5. Based on this table, most of the variables had VIF values of less than 5, indicating that the obtained model has statistical significance. To examine the relative importance, as well as the contribution of each descriptor in the model, the value of the mean effect (MF) was calculated for each descriptor. This calculation was performed using the following equation.

$MF_{j}=\frac{\beta \sum_{i=1}^{i=n}\mathrm{dij}}{\sum_{j}^{m}\mathrm{\beta j}\sum_{i}^{n}\mathrm{\beta ij}}$                     (3)

Where *MF*_j_ epresents the mean effect for the considered descriptor *j, βj* is the coefficient of the descriptor *j, dij* tands for the value of the target descriptors for each molecule and eventually, *m *is the descriptors number for the model. The MF value indicates the relative importance of a descriptor, compared with the other descriptors in the model. Its sign (+, -) indicates the variation direction in the values of the activities as a result of the increase (or decrease) in the descriptor values. The mean effect values are shown in Table 5.

**Table 4:** The correlation coefficient existing between the variables used in different GA-MLR

	BELm6	E1m	E2v	HATS8m	R2e+
BELm6	1	0	0	0	0
E1m	-0.32678	1	0	0	0
E2v	0.023974	-0.20311	1	0	0
HATS8m	-0.59	0.688288	-0.16102	1	0
R2e+	-0.19761	0.223311	-0.35277	0.508854	1

**Table 5:** The linear model based on seven parameters selected by the GA-MLR method

Descriptor	Chemical meaning	MF^a^	VIF^b^
Constant	Intercept	0	0
BELm6	lowest eigenvalue n. 6 of Burden matrix / weighted by atomic masses	0.746010645	1.47149272
E1m	1st component accessibility directional WHIM index / weighted by mass	-0.138362847	1.889935834
E2v	2nd component accessibility directional WHIM index / weighted by van der Waals volume	-0.259229493	1.062263659
HATS8m	leverage-weighted autocorrelation of lag 8 / weighted by mass	0.22801657	3.166979174
R2e+	R maximal autocorrelation of lag 2 / weighted by Sanderson electronegativity	0.423565125	1.500297586

^a ^Mean effect ^b ^Variation inflation factors

## Conclusion

In this article, a QSAR study of 36 molecules showing L-type calcium channel blocking activity was performed based on the theoretical molecular descriptors calculated by the DRAGON software. The built model was assessed comprehensively (internal and external validation) and all the validations indicated that the QSAR model built was robust and satisfactory and that the selected descriptors could account for the structural features responsible for the 1, 4 DHPs. The QSAR model developed in this study can provide a useful tool to predict the activity of new compounds and also to design new compounds with high calcium channel blocking activity. 
